# Glucocorticoid reduction in Glomerular Diseases

**DOI:** 10.1016/j.ekir.2026.103796

**Published:** 2026-01-24

**Authors:** Michael Toal, Mark Canney, Caitlin Hesketh, Todd Fairhead, David Massicotte-Azarniouch

**Affiliations:** 1 Division of Nephrology, University of Ottawa, Ottawa, Ontario, Canada; 2 Kidney Research Centre, Ottawa Hospital Research Institute, Ottawa, Ontario, Canada

**Keywords:** glomerular diseases, glomerulonephritis, glucocorticoid

## Abstract

Glucocorticoids (GCs) remain central to the management of many glomerular diseases, providing rapid and potent antiinflammatory effects. However, their well-recognized toxicities have driven growing efforts to reduce cumulative exposure. This review synthesizes evidence from randomized and key noncontrolled studies evaluating GC-sparing strategies across diseases where these agents have historically played a central therapeutic role. In antineutrophil cytoplasmic antibody (ANCA)-associated vasculitis (AAV), large trials such as the Plasma Exchange and Glucocorticoids in Severe ANCA-Associated Vasculitis (PEXIVAS) trial and the Effect of Reduced-Dose versus High-Dose Glucocorticoids Added to Rituximab (LoVAS) trial demonstrate that reduced-dose regimens preserve efficacy while lowering infection risk. In lupus nephritis (LN), lower-dose strategies and early use of multipronged regimens incorporating targeted agents such as belimumab, calcineurin inhibitors, or obinutuzumab support faster tapering. In minimal change disease (MCD), combination approaches with calcineurin inhibitors or mycophenolate achieve remission with less steroid exposure. In IgA nephropathy (IgAN), practice is increasingly shifting away from GC use altogether, as novel immunomodulatory agents targeting disease-specific pathways are integrated into care. Although GCs will likely remain essential for induction in select inflammatory glomerulopathies, their role can increasingly be narrowed to early, time-limited use. Future studies should address key uncertainties, particularly the optimal use of i.v. methylprednisolone in rapidly progressive presentations and evaluate the safety and efficacy of even lower-dose regimens.

## Graphical abstract

**Figure:**
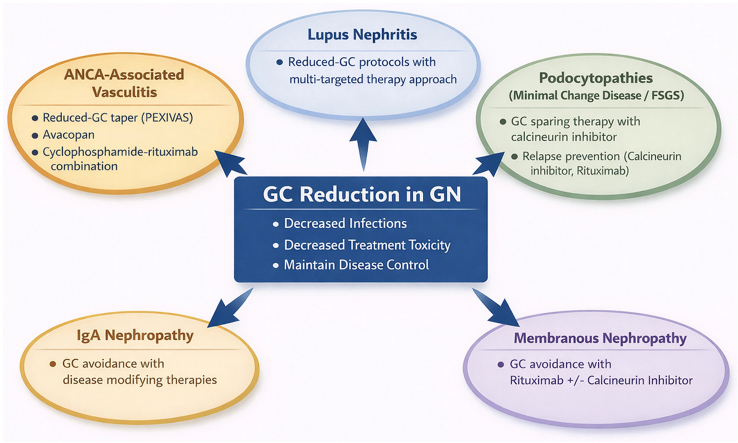

GCs have long been an essential component of treatment for many types of glomerular diseases. Although their efficacy in providing disease control and symptom relief is often undeniable, their use may be associated with significant side effects and toxicities. Consequently, there has been significant interest in developing strategies to reduce GC exposure in the management of glomerular diseases, aiming to minimize treatment-related adverse effects and improve the quality of life for patients without compromising efficacy in disease control.

This review examines the various strategies to reduce GC exposure in the management of glomerular diseases. The focus is on diseases where GC historically and continue to occupy a prominent role in management. A synthesis of evidence from major randomized controlled trials (RCTs) and observational studies that have examined GC-sparing strategies is provided by the type of glomerular disease. The objective of this review is to provide a better understanding of the role GCs play in the management of glomerular diseases and how to balance risks versus benefits. Ultimately, this can guide clinicians on the optimal use of GC and GC-sparing strategies.

To inform this narrative review, a systematic review of the literature was conducted to identify RCTs where a GC-sparing therapy was examined. The search was conducted in Ovid Medline, Embase, and Cochrane from inception to March 25, 2025, and focused on specific glomerular diseases (AAV, IgAN, LN, membranous nephropathy, minimal change disease [MCD]/nephrotic syndrome [NS], focal segmental glomerulosclerosis [FSGS], and antiglomerular basement membrane disease). To be eligible for inclusion, the purpose of the RCT had to be to reduce cumulative GC exposure in one of the arms, either through lower dose or duration of treatment or with GC-sparing therapy. Studies reported in English and conducted in pediatric or adult populations were included. The review was registered on PROSPERO (ID 1067595), and the search strategy is available in the Supplementary File S1. The search yielded 1502 studies. Two reviewers independently screened titles and abstracts and subsequently reviewed full texts of 77 studies for eligibility. Thirty-five studies (total patients = 3352) were included (Supplementary Figure S1). The details are presented in Supplementary Table S1 and references on these studies are presented in the Supplementary References). Selected studies from this systematic review, along with key published noncontrolled studies, inform the narrative review provided here on GC-sparing therapies in glomerular diseases.

### AAV

GCs have been a mainstay of treatment for AAV since the 1950s.^1^^,^^2^ Their use has long been dictated by expert opinion rather than experimental data.^3^ Though effective in providing symptom relief, it was recognized that prolonged use at high doses leads to significant treatment-related toxicity. Therefore, treatment regimens aiming to limit the use of GCs could improve patient outcomes.

To date, 2 RCTs have directly examined GC reduction, without the use of a GC-sparing agent, in AAV: the PEXIVAS trial and the LoVAS trial. PEXIVAS was a large (*N* = 704) trial which examined a reduced-dose GC tapering regimen, equating to an approximately 40% reduction in cumulative dose compared with the standard-of-care. The trial was highly enriched in patients with severe kidney involvement (mean serum creatinine: 330 μmol/l, 20% requiring acute dialysis), at highest risk for adverse outcomes. Patients received induction cyclophosphamide [CYC] (84%) or rituximab [RTX] (16%) alongside the GC taper. Reduced-GC was noninferior for the primary outcome of end-stage kidney disease (ESKD) or death (absolute risk difference: 2.3%; 95% confidence interval: −4.5 to 9.1), there was no significant difference in change in estimated glomerular filtration rate (eGFR) from baseline; however, there was a significant reduction in serious infections.^4^^,^^5^ The findings from PEXIVAS helped establish a guideline-recommended standard-of-care for prednisone use in severe AAV.^6^^,^^7^ The LoVas trial (*N* = 140) examined an even greater reduction of GC than PEXIVAS, but in patients with moderate severity AAV, and on a background induction of RTX in all patients.^8^ There was no difference in remission rates; however, there were less infections. LoVAS was conducted in Japanese patients with a predominance of antimyeloperoxidase ANCA positivity; therefore, generalizability may be limited. However, taken together, these trials demonstrate that reduction of GC in AAV is possible without compromising efficacy. A systematic review of reduced-GC dosing in AAV included these 2 trials and concluded that reduced-GC regimens probably decrease serious infections, may reduce risk of death, while probably having little or no effect on ESKD, remission, and relapse.^9^ Although much emphasis is put on reducing GC-related side effects, reduction of infections with reduced GC is not to be neglected because infection is the predominant cause of mortality.^10^^,^^11^ When comparing infection reduction achieved in PEXIVAS and LoVAS, it can be hypothesized that reducing GC exposure reduced the risk of infection (Table 1). Further reduction in GC exposure could lead to even greater reduction in infections.

**Table 1:** GC reduction and infection rates in AAV trials

Study	Cumulative oral GC-use (prednisone)	GC reduction	Infection rates (intervention vs. control)	Infection risk reduction
PEXIVAS (2020)	Received GC (median) Intervention: 2310 mg Control: 3990 mg	42%	Serious infections: 27.2% vs. 33.0%	IRR 0.69 à 31%
LOVAS (2021)	Received GC (median; at 6 mos) Intervention: 1318 mg Control: 4151 mg	68%	Serious infections: 7.2% vs. 20.0% Any infection: 15.9% vs. 44.6%	RR 0.36 à 64%^a^ RR 0.36 à 64%^a^
ADVOCATE (2021)	Received GC (median; at 6 mos) Intervention: 914 mg Control: 3124 mg	71%	Serious infections: 13.3% vs. 15.2% Any infection: 68.1% vs. 75.6%	RR 0.88 à 12%^a^ RR 0.90 à 10%^a^

AAV, antineutrophil cytoplasmic antibody–associated vasculitis GC, glucocorticoid; IRR, incidence rate ratio; RR relative risk.

a Calculated based on number of events provided in the publication.

Organ-threatening AAV is typically treated with high dose pulses of i.v GC for 1 to 3 days. The rationale is to achieve more rapid onset through nongenomic effects on GC receptors.^12^^,^^13^ A few observational, retrospective studies examined the use of pulsed methylprednisolone compared with no pulses in AAV with severe kidney involvement, or of reduced-dose pulsed methylprednisolone and prednisone compared with standard-doses.^14^^,^^15^ These studies did not demonstrate a clear benefit from i.v. methylprednisolone or higher doses; however, it is impossible to draw conclusions because of inherent limitations in study designs. A dedicated RCT would be informative given potential toxicity from high doses of iv. GC. Until then, judicious use of lower (500 mg/dose) and fewer doses might be reasonable.

Although RCTs support the use of reduced-dose prednisone regimens in AAV, there may be reservations in clinical practice. Real-world evidence from use of PEXIVAS-like reduced GC has shown conflicting results. In patients treated at vasculitis centers in France, a reduced-dose strategy was associated with worse outcomes.^16^ A single-center study from Ontario, Canada found that the adoption of the PEXIVAS reduced-dose GC taper was not associated with worse outcomes.^17^ These disparate findings may relate to differences in outcome definitions and patient populations. This Canadian study’s patient population had severe kidney involvement and similar baseline characteristics to the PEXIVAS trial population. The primary outcome of ESKD or death, as in PEXIVAS, was not significantly different between reduced-GC and standard-GC tapers (adjusted hazard ratio: 0.92 [0.42–2.00]). The French study had a composite of disease progression, major relapse, minor relapse, ESKD, or death as the primary outcome, where reduced-GC was not significantly associated with any individual outcomes. In addition, patients with more severe disease may not do as well with rapid reduction in GC if RTX is the only induction agent. In PEXIVAS, the subgroup of RTX-treated patients had a nonsignificantly increased risk for adverse outcomes in the reduced-GC arm, as did the subgroup in LoVAS where baseline eGFR was < 50 ml/min.^4^^,^^8^^,^^16^ This may raise concerns with the use of reduced GC in patients with most severe forms of disease on a background of RTX-only induction; an important consideration because in many centers, RTX is the induction agent of choice over CYC.

The combination of RTX and CYC offers a potential strategy to safely reduce GC in severe AAV, with even greater GC reduction than in previous RCTs. Observational studies have demonstrated that combining RTX with lower dose or shorter duration CYC allows for drastic reductions in GC use.^18^, ^19^, ^20^, ^21^ A group from the United Kingdom and Ireland published experience on 1 to 2 weeks of GC use with RTX-CYC combination in severe AAV. They demonstrated high rates of remission with very few requiring reintroduction of GC, and lower rates of infections than historical controls.^21^ These promising results offer a potentially safe, effective, and widely available GC-sparing therapy. However, such a regimen needs to be examined in a randomized controlled fashion; a clinical trial is currently on-going (NCT06983821).

The complement pathway has emerged as an important target in the pathophysiology of AAV, and to provide GC-sparing therapy in AAV. The C5a Receptor Inhibitor Avacopan in ANCA-Associated Vasculitis phase 2 trial first demonstrated that avacopan significantly reduced GC exposure while maintaining adequate disease control.^22^ The large phase 3 Avacopan for the Treatment of ANCA-Associated Vasculitis (ADVOCATE) trial established the efficacy of avacopan as a GC-sparing therapy in AAV.^23^ With GC removal within 4 weeks of starting avacopan during induction therapy (leading to a 71% reduction in prednisone exposure compared with placebo), avacopan was noninferior to a prednisone taper for remission at 26 weeks, superior for remission at 52 weeks, showed greater improvement in eGFR in patients with baseline eGFR < 30 ml/min, and was associated with significantly less GC-related side effects. Although encouraging, there are limitations to highlight. In ADVOCATE, participants in the placebo arm induced with RTX (about two-thirds of trial participants) did not receive RTX maintenance and had GC entirely removed at 21 weeks, with no further therapy until 52 weeks. This would not be considered current standard-of-care because RTX is typically given 4 to 6 months for maintenance therapy. Therefore, the superiority for sustained remission must be taken with reservations, particularly given that the difference in sustained remission was mostly observed in the subgroup of RTX-induced patients (56% vs. 71% for prednisone vs. avacopan, respectively) and not those who received CYC induction with azathioprine maintenance (53% vs. 56% for prednisone vs. avacopan, respectively). In addition, despite this level of GC reduction, there was only a modest effect on reducing infection, less than would be expected based on the findings from PEXIVAS and LoVAS (Table 1). Therefore, though GC-sparing, avacopan may not resolve the important issue of high infection rates in patients with severe AAV. Further, avacopan’s safety and efficacy have yet to be firmly established in patients with most severe forms of AAV (eGFR < 15 ml/min or alveolar hemorrhage requiring invasive ventilation) because they were excluded from ADVOCATE. Furthermore, the cost and availability of avacopan may limit its more widespread use for many patients throughout the world. Concerns regarding liver toxicity with avacopan have been raised and in ADVOCATE was reported in 5% of participants in the experimental group. Observational studies have demonstrated much higher rates in Japanese populations (17%–44%),^24^^,^^25^ whereas rates in non-Japanese observational studies were similar to those in ADVOCATE (3%–4%).^26^^,^^27^ It is unclear if there are genetic predispositions to liver toxicity from avacopan. Other GC-sparing complement pathway blockers are under study at the time of writing. Vilobelimab, a monoclonal antibody against the C5a receptor, has data from a phase 2 trial demonstrating GC-sparing ability.^28^ The greater understanding of the complement pathway in the pathogenesis of AAV shows promise in offering GC-sparing therapies. However, further studies are needed to clarify their impact on infection rates and their efficacy and safety in patients with most severe forms of AAV, often at greatest risk for infections.

Low-dose prednisone (< 10 mg/d) has historically been an important component of maintenance therapy. A 2010 meta-analysis compared patients from RCTs where patients had stopped GC entirely at the end of follow-up compared with low-dose maintenance. A higher relapse rate was noted with GC removal (43%) than with GC continuation (14%).^29^ However, this meta-analysis included studies conducted in an era when RTX was not readily available as maintenance therapy. The TAPIR trial (not yet published; preliminary data presented at conference meetings) examined prednisone removal compared with prednisone 5 mg/d in patients with granulomatosis with polyangiitis in remission within 1 year of induction therapy. There were more relapses with prednisone removal (16%) compared with 5 mg/d (4%). However, relapses were almost exclusively minor, rates were low and were similar in the subgroup of patients who received RTX maintenance (9% vs. 6% for removal vs. 5 mg/d, respectively).^30^ Therefore, if RTX is available, the selected maintenance agent, low-dose prednisone for maintenance therapy may not be necessary, even in patients typically deemed at higher risk of relapse.

### LN

Systemic lupus erythematosus is a multisystem autoimmune disease with a 20% to 60% risk of LN and a high lifetime risk of ESKD in patients with proliferative forms of LN.^31^ Short-term treatment outcomes improved dramatically with GC treatment in the 1950’s; however, poor long-term outcomes have led to a standard-of-care with GC and additional immunosuppression for induction of disease remission followed by prolonged maintenance therapy.^32^^,^^33^ Despite advances in systemic lupus erythematosus pathophysiology and immunosuppression, GCs remain a mainstay of therapy. Considering that this is a chronic incurable condition associated with relapses of activity which often affects younger adults, the lifetime GC burden can be significant, therefore GC-sparing strategies are important to consider.

A systemic review and meta-analysis of the control arms of randomized clinical trials evaluated the effect of GC regimens on kidney response, infections and mortality in patients with LN.^34^ The authors analyzed 3231 patients in 50 RCTs and found that an initial GC dose of 60 mg/d without pulses, compared with 25 mg/d, led to improved complete renal remission (34.6% vs. 19.5%) at the expense of increased serious infections (12.1% vs. 3.2%) and death (2.7% vs. 0.2%). The addition of initial GC pulses increased the rates of complete remission and death but not serious infections. Based on this systematic review, it is unclear where the ideal starting dose lies, balancing the trade-off between disease control and treatment-related toxicity. Although the dose and duration of GC pulse have not been formally examined, the specific impact of oral GC reduction for induction therapy has directly been examined in a few small studies (Table 2). In an international study, 81 participants were randomized to divergent prednisone regimes after 3 pulses of methylprednisolone, and with concomitant mycophenolate. The average cumulative doses were 4630 mg in the reduced-GC group compared with 7111 mg in the control. Rates of complete remission at 24 weeks were equivalent between the 2 groups (20.5% reduced GC vs. 19.0% standard GC); however, there were less adverse events in the reduced GC-group (19% vs. 10%, respectively).^35^ Bandhan *et al.*^36^ examined a reduced-GC induction regimen (prednisone starting dose 0.5 mg/kg with rapid taper compared with 1 mg/kg) in 27 Bangladeshi patients. Both groups received pulse i.v. CYC and oral hydroxychloroquine. At 24 weeks, there was a lower cumulative GC dose (6055 mg vs. 2205 mg) with equivalent complete renal remission rates (both 66.7%) but less serious infections (2 vs. 4 events).^36^ Bharati *et al.*^37^ studied a reduced GC-starting dose (0.5 mg/kg vs. 1 mg/kg starting prednisone dose) with a quicker taper in conjunction with mycophenolate mofetil (MMF), renin-angiotensin-aldosterone system inhibition and hydroxychloroquine in 20 Indian patients. This trial was terminated early because an interim analysis demonstrated worse remission rates in the low-dose group (10%) compared with the standard-dose group (70%) by week 24. This group did however achieve a lower cumulative GC dose of almost half at 24 weeks (3210 mg vs. 6450 mg) with more GC-related adverse events noted in the control group.^37^ Observational study protocols have described avoidance of oral GCs (only using i.v. methylprednisolone pulses) combining RTX and mycophenolate for induction treatment of LN.^38^ The Rituximab and Mycophenolate Mofetil Without Oral Steroids for Lupus Nephritis trial was meant to properly examine this question; however, the trial was terminated without any published results at this time. There is currently insufficient data to confirm the safety and efficacy of prednisone avoidance during induction therapy.

**Table 2:** Summary of steroid-sparing trials in lupus nephritis

Author (year, location)	Duration	Primary outcome	Intervention	Comparator	Key findings
Zeher (2011, International)	24 wks	CR UPCR < 0.5 with normalized urine sediment and serum creatinine within 10% of baseline value	Weight-based prednisolone starting at 22.5–35 mg/d reducing to 2.5–5 mg/d by week 24 (*n* = 39)	Weight-based prednisolone starting at 45–70 mg/d reducing to 5–10 mg/d by week 24 (*n* = 42)	Equivalent rates of CR (20.5% reduced dose vs. 19% standard dose.) Increased infection rates in standard dose (57%) compared with reduced dose (36%)
Bharati (2019, India)	24 wks	CR (> 50% reduction of proteinuria and stabilization of creatinine)	Prednisone 0.5 mg/kg/d for 8 wks, then reduced by 0.1 mg/kg every 4 wks to 0.1 mg/kg maintenance (*n* = 10)	Prednisone 1 mg/kg/d for 8 wks, then reduced by 0.2 mg/kg every 4 wks to 0.1 mg/kg maintenance (*n* = 10)	CR rates higher in standard dose (70%) regimen compared with the reduced dose (10%). More infections in standard-dose group (30% vs. 0%)
Bandhan (2021, Bangladesh)	24 wks	CR (UPCR < 500 mg/d and return to baseline creatinine)	Prednisone 0.5 mg/kg/d for 4 wks, tapered by 5 mg/wk until 10 mg, then 2.5 mg to 7.5 mg maintenance (*n* = 15)	Prednisone 1 mg/kg/d for 4 wks, tapered by 10 mg every 2 wks to 40 mg, then 5 mg every 2 wks to 10 mg, then tapered by 2.5 mg to maintenance 7.5 mg (*n* = 12)	Complete renal remission achieved in 66.7% in each group No significant difference in adverse events

CR, complete remission; KRT, kidney replacement therapy; UPCR, urine protein-to-creatinine ratio.

More recently, large international clinical trials have used lower doses of induction GCs with novel immunosuppressive therapies or combination immunosuppression. The Efficacy and Safety of Obinutuzumab in Active Lupus Nephritis trial studied obinutuzumab as an add-on therapy to mycophenolate and prednisone and the Safety and Efficacy of Long-Term Voclosporin Treatment for Lupus Nephritis trial studied the calcineurin-inhibitor, voclosporin, as add-on to mycophenolate and prednisone.^39^^,^^40^ Although neither reported the cumulative GC dose, the protocolized prednisone taper in both arms of these trials started at lower doses and achieved low maintenance dose (< 5 mg/d) more quickly than historical large induction trials in LN such as the Aspreva Lupus Management Study Group trial.^41^ Although this does not provide a direct comparison of reduced-GC regimens, it suggests that in the current landscape of upfront multitargeted therapy, lower prednisone regimens can be used. This should be done with the doses of coinduction agents typically studied in RCTs and recommended in clinical guidelines. The updated 2024 Kidney Disease: Improving Global Outcomes clinical practice guidelines include a practice point to use reduced-dose GC following a short course of methylprednisolone pulses during initial treatment of active LN.^31^ The optimal dose of GC in the initial therapy remains unanswered and there is an opportunity for differential GC doses to be included in clinical trials. There is uncertainty regarding the role of GCs in maintenance treatment of LN because this has only been examined in a small pilot trial and in systemic lupus erythematosus with nonkidney involvement, with differing results.^42^^,^^43^ Clinicians should be guided by the unique balance of risks for each patient to help manage the short and long-term sequelae of this condition.

### MCD and NS

The rapid effect and high remission rates with GC in MCD or NS are the perfect example of the potential efficacy of GC for management of autoimmune disease and make them the benchmark for treatment of MCD. However, many studies have examined the use of shorter duration and lower doses of GC, mostly in the pediatric population (Supplementary Table S1). It is worth highlighting that prednisone use in MCD or NS is one of the rare areas of nephrology where evidence base is more robust in pediatric than in adult nephrology. To our knowledge, there are currently no RCTs in adults which have examined the optimal dose and duration of GC use for MCD or NS. A Japanese, observational before-and-after study examined the prospective use of a short GC taper like what is used in pediatrics (8–10 weeks) compared with a historical standard GC taper of 1 to 2 years in adults with a first episode of MCD or NS who achieved complete remission. The short GC group had earlier occurrence of relapse; however, there was no difference in the number of frequent relapsers and the cumulative GC exposure was significantly lower in the short GC group.^44^ Although this suggests that shorter duration of GC could be used, most of the research in adult MCD or NS has focused on examining the safety and efficacy of GC-sparing therapies used up front such as calcineurin inhibitors and MMF.

#### Calcineurin Inhibitors

Calcineurin inhibitors do not simply lower proteinuria in MCD through hemodynamic decrease in glomerular filtration pressure, but more importantly through immunosuppressive effects on T cells and even direct podocyte stabilizing effects.^45^ Therefore, calcineurin inhibitors are important treatment options and both tacrolimus and cyclosporine have been evaluated in RCTs as GC-sparing strategies for the treatment of new or relapsed MCD or NS. In most of these trials, the drug was started immediately at the onset of disease and prescribed concurrently with reduced-dose prednisone (Table 3). The studies by Eguchi *et al.*^46^ and Miao *et al.*^47^ suggested similar rates of remission with this approach; however, these trials had limitations, including small sample size, lack of a power calculation, and suboptimal reporting of adverse events. In a more recent study, Chin *et al.*^48^ tested noninferiority of tacrolimus 0.05 mg/kg twice daily plus once daily prednisone 0.5 mg/kg/d compared with prednisone 1 mg/kg/d in patients with new or relapsing disease (PMID 33168602). The primary outcome was complete remission defined as urine protein-to-creatinine ratio < 0.2 g/g within 8 weeks. In the primary efficacy analysis, 53 of 67 patients (79.1%) in the tacrolimus and low-dose steroid arm and 53 of 69 patients (76.8%) in the high-dose steroid arm achieved complete remission. The upper confidence limit (11.6%) was below the prespecified noninferiority margin of 20%, thus establishing noninferiority of the intervention. Patients receiving tacrolimus achieved remission faster (median: 15 vs. 25 days) and had a lower rate of relapse (5.7% vs. 22.6%). The total number of adverse events was similar in both groups. Patients in the high-dose GC arm experienced more infections whereas patients in the tacrolimus arm had more gastrointestinal symptoms.

**Table 3:** Summary of randomized controlled trials examining GC-sparing strategies for treating nephrotic syndrome due to minimal change disease in adult patients

Author (year, location)	Duration	Primary outcome	Intervention	Comparator	Key findings
Calcineurin inhibitor					
Miao (2006, China)	24 wks	CR (proteinuria < 0.3 g/d) or PR (proteinuria 0.3–2.9 g/d and serum albumin ≥ 30 g/l)	Tacrolimus 2 g/d plus prednisone 30 mg/d (*n* = 30)	Prednisone 1 mg/kg/d to a max of 60 mg/d (*n* = 30)	All patients achieved CR/PR in both groups CR achieved in 29 (97%) patients in intervention and 27 (90%) in control Less weight gain in intervention group
Eguchi (2010, Japan)	24 wks	CR (proteinuria < 0.3 g/d)	Cyclosporine to target C2 level 600–800 ng/ml (mean dose: 1.8 mg/kg/d) plus prednisone 0.8 mg/kg/d (*n* = 26)	Prednisone 1 mg/kg/d (*n* = 26)	Numerically higher CR rate in cyclosporine/prednisone group at 4 wks (96% vs 77%) Similar rates of CR at 3 mos and 6 mos in both groups Faster mean time to remission in intervention group (13 d vs. 20 d) No reporting of AEs
Li (2017, China)	64 wks	CR (proteinuria < 0.3 g/d) or PR (proteinuria < 3.5 g/d but > 0.3 g/d)	i.v. MP 0.8 mg/kg/d for 10 d followed by tacrolimus 0.05 daily (*n* = 63)	i.v. MP 0.8 mg/kg/d for 10 d followed by prednisone 1 mg/kg/d (*n* = 56)	Similar remission rates in both groups (98% in intervention and 96% in control) Tacrolimus was noninferior to prednisone Similar time to remission in both groups Similar relapse rate in both groups More SAEs in prednisone arm (7 vs. 2)
Medjeral-Thomas (2020, UK)	Median 44 wks	CR at 8 wks (UPCR < 50 mg/mmol)	Tacrolimus 0.1 mg/kg/d (*n* = 27)	Prednisone 1 mg/kg/d to a max of 60 mg daily (*n* = 25)	Lower remission rate in tacrolimus group (63%) than in prednisone (84%) Similar rates of relapses in both groups Similar rates of AEs
Chin (2021, South Korea)	24 wks	CR within 8 weeks (UPCR <0.2 g/g)	Tacrolimus 0.1 mg/kg per day plus prednisone 0.5 mg/kg per day (n=67)	Prednisone 1 mg/kg per day (n=69)	Similar CR rate in tacrolimus/prednisone (79%) and high-dose prednisone (77%) Fewer relapses observed in intervention group (5.7% vs. 22.6%) Similar rate of AEs in both groups

AE, adverse event; CR, complete remission; MP, methylprednisolone; PR, partial remission; SAE, serious AE; UPCR, urine protein-to-creatinine ratio.

In another noninferiority study, Li *et al.*^49^ evaluated sequential treatment with tacrolimus after a short course of corticosteroids compared with prednisone monotherapy. All patients received 10 days of i.v. methylprednisolone at the time of diagnosis. Thereafter, patients allocated to the intervention arm received tacrolimus 0.05 mg/kg/d and patients in the active comparator arm received prednisone at an initial dose of 1 mg/kg/d. Tacrolimus was found to be noninferior to prednisone. Over the course of 64 weeks, rates of complete or partial remission were very high in both groups (98% and 96%, respectively). Time to remission and rates of relapse were similar in both groups; however, patients receiving prednisone experienced a higher frequency of serious adverse events.

Few studies have evaluated the efficacy and safety of tacrolimus monotherapy without any upfront GC for treatment of MCD. In a multicenter open-label RCT, Medjeral-Thomas *et al.*^50^ recruited 55 adult patients with *de novo* disease who were allocated to receive either tacrolimus 0.05 mg/kg twice daily or prednisolone 1 mg/kg/d to a maximum dose of 60 mg/d. The primary outcome was the rate of complete remission (defined as urine protein-to-creatinine ratio < 50 mg/mmol) after 8 weeks of treatment. Although the remission rates were not statistically significant between the groups, more patients in the prednisone arm achieved remission (85% vs. 63%) and the investigators thus failed to show noninferiority of tacrolimus therapy. Secondary analyses of remission rates at 16 and 24 weeks indicated that progressively more patients in the tacrolimus arm achieved remission, suggesting that there was a delayed response to therapy. Relapses were common in both groups (17/23 in the steroid group; 16/22 in the tacrolimus group) and most relapses in both groups occurred after therapy had been discontinued. The frequency of adverse events and serious adverse events was similar in both groups. These findings suggest that tacrolimus may not be an appropriate treatment strategy if rapid control of NS is needed. For example, the complete remission rate at 4 weeks was just 22% in the tacrolimus group compared with 64% in the prednisone group.

The totality of evidence suggests that a combination approach using tacrolimus and reduced-dose prednisone is noninferior to high-dose prednisone in achieving complete remission, has a favorable safety profile, and may reduce the frequency of relapse when the medications are given concurrently. A treatment strategy using tacrolimus without any GC is likely to result in a longer time to remission, and it is unclear to what extent it could benefit patients in terms of less adverse events or lower likelihood of relapse.

#### MMF

Fewer studies have investigated MMF as a treatment for MCD (Table 3). The largest clinical trial was conducted in France and recruited 116 patients with biopsy-proven MCD who were randomly allocated to receive enteric-coated mycophenolate sodium 720 mg twice daily plus prednisone 0.5 mg/kg/d (intervention) or high dose prednisone 1 mg/kg/d with a maximum dose of 80 mg/d (comparator).^51^ The primary outcome was the proportion of patients who achieved complete remission at 4 weeks. There was no difference in the remission rate between the groups at 4 weeks (61.5% in MMF group and 57.9% in control group) or at later time points up to 52 weeks. There were numerically less relapses in the MMF group (9 vs. 6); however, there was no statistically significant difference between the groups in relapse-free survival. A total of 18 serious adverse events were recorded during the trial, 9 in each group. Similar findings were demonstrated in a subsequent smaller trial conducted in China in which 8 out of 10 patients achieved CR after 24 weeks of therapy with MMF and low dose prednisone, compared with 7 out of 10 patients who were treated with high-dose prednisone monotherapy.^52^ These findings suggest that combination therapy with MMF and low-dose prednisone has similar efficacy to high-dose prednisone monotherapy, although the evidence base is limited.

#### RTX

There is limited data to support the use of RTX as a first-line induction agent for an acute episode of NS from MCD. Most studies have examined RTX to prevent relapse in patients with frequently-relapsing or steroid-dependent NS. In this context, multiple observational and noncontrolled studies, as well as a Japanese RCT have demonstrated its efficacy to maintain remission.^53^, ^54^, ^55^, ^56^ In the double-blind placebo-controlled RCT by Isaka *et al.*,^57^ 72 patients who had achieved remission were randomized to RTX or placebo. At week 49, RTX led to greater relapse-free survival (87% vs. 38%), a higher proportion of patients discontinuing corticosteroid (72% vs. 36%), and a longer time to relapse (49 weeks vs. 31 weeks). These results may alter the way relapse prevention is approached in MCD, where RTX is available. Future studies should examine the role for first-line use of RTX with a shortened course of prednisone to rapidly and effectively induce durable remission of acute NS, which could represent a major step for GC-sparing therapy in MCD.

### FSGS

FSGS is a histopathologic pattern rather than a single disease entity and encompasses a heterogeneous group of clinical phenotypes with distinct pathogenic mechanisms and therapeutic implications. Patients with presumed primary FSGS, typically defined by full NS and diffuse podocyte foot process effacement on electron microscopy, are commonly managed as immune-mediated podocytopathy. In this context, high-dose oral GC remain the recommended first-line therapy, even though there are no RCTs directly demonstrating their efficacy in FSGS.^58^ Compared with MCD, FSGS is more frequently resistant to therapy, and calcineurin inhibitors are recommended as alternative first-line agents or in cases of GC resistance or intolerance. Observational data suggest that RTX may be considered in difficult to treat patients.^53^ Evidence supporting upfront GC-sparing strategies in FSGS are limited. One small, single-center pilot trial from India evaluated MMF combined with reduced-dose prednisone (0.5 mg/kg/d for 2–3 months) versus standard-dose prednisone in patients with NS due to membranous nephropathy or FSGS.^59^ Among the 33 patients with FSGS, MMF with reduced-dose prednisone achieved similar efficacy while reducing cumulative GC exposure by approximately 74%. However, the modest sample size, mixed disease population, and lack of subsequent replication limit the generalizability of these findings and likely explain why this approach has not been widely adopted in clinical practice. Genetic testing is increasingly central to the management of FSGS, particularly by identifying patients less likely to respond to immunosuppression mitigating unnecessary treatment toxicity. Genotype-informed approaches may enable targeted therapies, exemplified by emerging agents such as inaxaplin for APOL1-mediated FSGS.^60^

### IgAN

The treatment landscape of IgAN has shifted to reduced-GC protocols and even foregoing them altogether. The Effect of Oral Methylprednisolone on Decline in Kidney Function or Kidney Failure in Patients With IgA Nephropathy trial was the largest study in IgAN, and although it did not directly compare different GC regimens, it demonstrated efficacy and safety of a reduced-GC regimen (oral methylprednisolone starting at 0.4 mg/kg/d), after an excess in serious infections was observed with the full-dose regimen (starting dose: 0.6–0.8 mg/kg/d) in the initial Effect of Oral Methylprednisolone on Decline in Kidney Function or Kidney Failure in Patients With IgA Nephropathy trial.^61^ When adverse events were compared between participants who had received the reduced-dose and the full-dose regimens, there were less serious adverse events in the reduced-dose, mostly driven by less serious infections (4% vs. 9%, respectively). A few studies directly examined GC-sparing strategies, either with i.v. methylprednisolone pulses, leflunomide, or mycophenolate and found similar efficacy, favorable safety, and lower cumulative GC exposure than with a standard GC regimen.^62^, ^63^, ^64^

Advances in the understanding of pathophysiological mechanisms has led to novel treatments, which may obviate the need for GC altogether.^65^, ^66^, ^67^ Such therapies are currently under study, and access is limited; however, these may change the landscape of IgAN treatment. A large Indian study is examining commonly available, generic medications (budesonide, mycophenolate, and hydroxychloroquine) in a multiarm platform trial (GRACE-IGAN NCT06676384). This could provide crucial data for clinicians and patients in jurisdictions where newer agents may not be available but alternatives to systemic GCs are preferred.

### Membranous Nephropathy

GC-based therapies have typically been reserved for most severe cases of membranous nephropathy, typically in alternating monthly schedules with a cytotoxic agent based on the 1992 protocol by Ponticelli *et al.*^68^ The need for and amount of GCs in the cyclical ponticelli-type regime is unclear because that specific aspect has never been properly examined. Alternative agents are available which forgo GCs entirely. Because of its more favorable safety profile, administration, and monitoring requirements, RTX monotherapy has been widely adopted as an efficacious therapy which avoids the use of GC. Calcineurin inhibitors, with or without low-dose GC, are another option, but not as effective as RTX.^69^ Nevertheless, CYC- or GC-based regimens are still used, particularly in patients with very high-risk disease, or when RTX is ineffective or unavailable.^70^ Furthermore, some have combined RTX and CYC or GCs in high-risk patients with low dose or short duration CYC and rapid prednisone taper.^71^^,^^72^ These regimens showed high remission and low rates of serious adverse events; however, their safety and efficacy need to be confirmed in prospective controlled studies.

### Conclusion

GCs remain a cornerstone in the management of several glomerular diseases, offering rapid, potent antiinflammatory effects and high efficacy unlikely to be surpassed in many conditions. However, the cumulative burden of GC toxicity necessitates more deliberate and evidence-informed use. The goal should not be complete elimination, but rather strategic, time-limited use early in the disease course when benefits are greatest and needed in certain highly inflammatory diseases, complemented by targeted, disease-specific therapies to minimize prolonged exposure. As the therapeutic landscape evolves with the introduction of novel immunomodulatory agents and biologics, reliance on GC should continue to diminish. Future studies should address key uncertainties, particularly the optimal use of i.v. methylprednisolone in rapidly progressive presentations and evaluate the safety and efficacy of even lower-dose regimens. Importantly, in many parts of the world, novel GC-sparing agents remain costly and often inaccessible, underscoring the need to optimize the use of widely available therapies such as GC themselves. Efforts to refine dosing strategies, duration, and combination approaches using accessible agents will therefore remain essential, ensuring that advances in care are both evidence-based and globally relevant.

## Disclosure

All the authors declared no competing interests.

## Supplementary Material

Supplementary File (PDF) **Supplementary References. File S1.** Search strategy for systematic review. **Figure S1.** Flow chart – Systematic review study selection on process. **Table S1.** Randomized controlled trials examining lower GC exposure compared to higher GC exposure in glomerular diseases.

## Footnotes

Supplementary File (PDF)

**Supplementary References.**
**File S1.** Search strategy for systematic review. **Figure S1.** Flow chart – Systematic review study selection on process. **Table S1.** Randomized controlled trials examining lower GC exposure compared to higher GC exposure in glomerular diseases.
