# Supplementary material for: Glucocorticoid reduction in Glomerular Diseases
Source: Kidney Int Rep. 2026 Jan 24;11(4):103796. doi: 10.1016/j.ekir.2026.103796 (PMC12925401; doi:10.1016/j.ekir.2026.103796)
Supplement: Supplementary File (PDF) — Supplementary References. File S1. Search strategy for systematic review. Figure S1. Flow chart – Systematic review study selection on process. Table S1. Randomized controlled trials examining lower GC exposure compared to higher GC exposure in glomerular diseases. [file mmc1.pdf]

Supplementary Appendix

File S1. Search strategy for systematic review

Ovid MEDLINE(R) ALL <1946 to March 25, 2025>

1 Anti-Neutrophil Cytoplasmic Antibody-Associated Vasculitis/ 3323
2 (anca adj3 vasculitis).tw,kf. 4998
3 Anti-Neutrophil Cytoplasmic Antibody-Associated Vasculitis.tw,kf. 524
4 IgA Vasculitis/ 5138
5 IgA vasculitis.tw,kf. 870
6 ((Allergic or Schoenlein Henoch or Rheumatoid or Nonthrombopenic) adj2 purpura).tw,kf. 721
7 Cryoglobulinemia/ 3399
8 Cryoglobulinemi*.tw,kf. 4003
9 Lupus Nephritis/ 8557
10 Lupus Nephritis.tw,kf. 11392
11 lupus Glomerulonephrit*.tw,kf. 292
12 Anti-Glomerular Basement Membrane Disease/ 2195
13 Anti-GBM Disease*.tw,kf. 581
14 (Goodpasture* adj (disease* or syndrome*)).tw,kf. 1379
15 Glomerulosclerosis, Focal Segmental/ 6120
16 Focal Segmental Glomerulosclerosis.tw,kf. 5364
17 Focal Sclerosing Glomerulosclerosis.tw,kf. 7
18 Focal Glomerulosclerosis.tw,kf. 587
19 Glomerulonephritis, Membranous/ 4283
20 Membranous Nephropath*.tw,kf. 5310
21 Membranous Glomerulonephri*.tw,kf. 1700
22 Nephrosis, Lipoid/ 2938
23 Minimal change disease*.tw,kf. 2332
24 nephrotic syndrome*.tw,kf. 22669
25 Minimal Change Glomerulonephri*.tw,kf. 120
26 Glomerulonephritis, IGA/ 7791
27 IgA nephropath*.tw,kf. 8996
28 IGA Glomerulonephri*.tw,kf. 350
29 immunoglobulin A nephropath*.tw,kf. 1626
30 or/1-29 72572
31 exp Adrenal Cortex Hormones/ 435814
32 Steroids/ad 3440
33 (corticosteroid* or Glucocorticoid* or Beclomethasone or Betamethasone or Budesonide or Clobetasol or Desoximetasone or Dexamethasone or Diflucortolone or Flumethasone or Fluocinolone Acetonide or Fluocinonide or Fluocortolone or Fluorometholone or Fluprednisolone or Flurandrenolone or Fluticasone-Salmeterol or Melengestrol Acetate or

Methylprednisolone or Paramethasone or Prednisolone or Prednisone or Tobramycin or Triamcinolone).tw,kf. 349562
34 steroid*.ti,kf. or steroid*.ab. /freq=2 145542
35 or/31-34 692511
36 30 and 35 14448
37 exp randomized controlled trial/ 636255
38 (random* or placebo).tw. or trial.ti. 1779052
39 37 or 38 1883016
40 exp animals/ not humans/ 5320986
41 protocol.ti. 95158
42 (exp child/ or exp infant/ or exp pediatrics/) not exp adult/ 2016045
43 ((child* or p?ediat* or infant or infants or newborn* or neonat*) not adult*).ti. 1483394
44 or/40-43 7798538
45 39 not 44 1546882
46 36 and 45 879

Embase Classic+Embase <1947 to 2025 March 25>

1 exp ANCA associated vasculitis/ 32028
2 (anca adj3 vasculitis).tw. 9714
3 Anti-Neutrophil Cytoplasmic Antibody-Associated Vasculitis.tw. 569
4 anaphylactoid purpura/ 9597
5 IgA vasculitis.tw. 1360
6 ((Allergic or Schoenlein Henoch or Rheumatoid or Nonthrombopenic) adj2 purpura).tw. 637
7 cryoglobulinemia/ 8100
8 Cryoglobulinemi*.tw. 6025
9 lupus erythematosus nephritis/ 24403
10 Lupus Nephritis.tw. 19411
11 lupus Glomerulonephrit*.tw. 418
12 Goodpasture syndrome/ 2681
13 Anti-GBM Disease*.tw. 992
14 (Goodpasture* adj (disease* or syndrome*)).tw. 1968
15 focal glomerulosclerosis/ 13672
16 Focal Sclerosing Glomerulosclerosis.tw. 12
17 Focal Glomerulosclerosis.tw. 729
18 membranous glomerulonephritis/ 11792
19 Membranous Nephropath*.tw. 8062
20 Membranous Glomerulonephri*.tw. 2441
21 lipoid nephrosis/ 2628
22 Minimal change disease*.tw. 3818
23 nephrotic syndrome*.tw. 32950

24 Minimal Change Glomerulonephri*.tw. 151
25 immunoglobulin A nephropathy/ 18204
26 IgA nephropath*.tw. 13651
27 IGA Glomerulonephri*.tw. 435
28 immunoglobulin A nephropath*.tw. 1944
29 or/1-28 138979
30 exp *corticosteroid/ or corticosteroid/ 649202
31 exp *steroid/ad, th 29033
32 (corticosteroid* or Glucocorticoid* or Beclomethasone or Betamethasone or Budesonide or Clobetasol or Desoximetasone or Dexamethasone or Diflucortolone or Flumethasone or Fluocinolone Acetonide or Fluocinonide or Fluocortolone or Fluorometholone or Fluprednisolone or Flurandrenolone or Fluticasone-Salmeterol or Melengestrol Acetate or Methylprednisolone or Paramethasone or Prednisolone or Prednisone or Tobramycin or Triamcinolone).tw. 550567
33 steroid*.ti. or steroid*.ab. /freq=2 202472
34 or/30-33 1058547
35 29 and 34 34523
36 random*.tw. or placebo*.mp. or double-blind*.tw. or trial.ti. 2622871
37 35 and 36 3072
38 (exp animals/ or animal experiments/ or nonhumans/) not exp humans/ 6649291
39 conference abstract.pt. 5421761
40 protocol.ti. 110862
41 (exp child/ or exp infant/ or exp pediatrics/) not exp adult/ 2800067
42 ((child* or p?ediat* or infant or infants or newborn* or neonat*) not adult*).ti. 1990901
43 or/38-42 14411720
44 37 not 43 1665

EBM Reviews - Cochrane Central Register of Controlled Trials <February 2025>

1 Anti-Neutrophil Cytoplasmic Antibody-Associated Vasculitis/ 147
2 (anca adj3 vasculitis).tw,kw. 535
3 Anti-Neutrophil Cytoplasmic Antibody-Associated Vasculitis.tw,kw. 20
4 IgA Vasculitis/ 72
5 IgA vasculitis.tw,kw. 18
6 ((Allergic or Schoenlein Henoch or Rheumatoid or Nonthrombopenic) adj2 purpura).tw,kw. 17
7 Cryoglobulinemia/ 39
8 Cryoglobulinemi*.tw,kw. 79
9 Lupus Nephritis/ 385
10 Lupus Nephritis.tw,kw. 1034
11 lupus Glomerulonephrit*.tw,kw. 16
12 Anti-Glomerular Basement Membrane Disease/ 8

13 Anti-GBM Disease*.tw,kw. 6
14 (Goodpasture* adj (disease* or syndrome*)).tw,kw. 12
15 Glomerulosclerosis, Focal Segmental/ 103
16 Focal Segmental Glomerulosclerosis.tw,kw. 216
17 Focal Sclerosing Glomerulosclerosis.tw,kw. 0
18 Focal Glomerulosclerosis.tw,kw. 99
19 Glomerulonephritis, Membranous/ 177
20 Membranous Nephropath*.tw,kw. 464
21 Membranous Glomerulonephri*.tw,kw. 181
22 Nephrosis, Lipoid/ 66
23 Minimal change disease*.tw,kw. 87
24 nephrotic syndrome*.tw,kw. 1522
25 Minimal Change Glomerulonephri*.tw,kw. 9
26 Glomerulonephritis, IGA/ 370
27 IgA nephropath*.tw,kw. 829
28 IGA Glomerulonephri*.tw,kw. 15
29 immunoglobulin A nephropath*.tw,kw. 424
30 or/1-29 4636
31 exp Adrenal Cortex Hormones/ 35808
32 Steroids/ad 0
33 (corticosteroid* or Glucocorticoid* or Beclomethasone or Betamethasone or Budesonide or Clobetasol or Desoximetasone or Dexamethasone or Diflucortolone or Flumethasone or Fluocinolone Acetonide or Fluocinonide or Fluocortolone or Fluorometholone or Fluprednisolone or Flurandrenolone or Fluticasone-Salmeterol or Melengestrol Acetate or Methylprednisolone or Paramethasone or Prednisolone or Prednisone or Tobramycin or Triamcinolone).tw,kw. 68474
34 steroid*.ti,kw. or steroid*.ab. /freq=2 15080
35 or/31-34 88537
36 30 and 35 2167
37 (clinical trial protocol or conference proceeding or trial registry record).pt. 891623
38 36 not 37 975

Figure S1. Flow chart – Systematic review study selection process

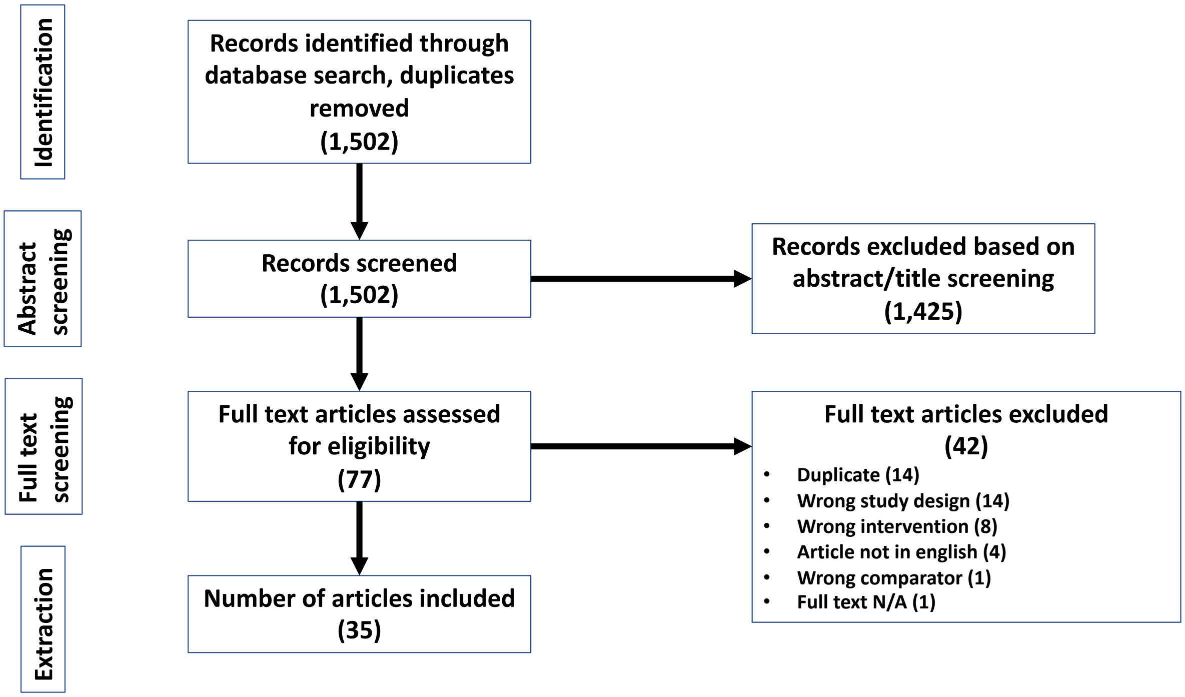

Table S1. Randomized controlled trials examining lower GC exposure compared to higher GC exposure in glomerular diseases

Study	Disease	GC reducing strategy
1988 Brodehl[S1]	MCD/NS (pediatric)	Shorter treatment
1988 Ueda[S2]	MCD/NS (pediatric)	2 months vs 8 months
1991 Tejani[S3]	MCD/NS (pediatric)	Cyclosporin with lower prednisone dose
1993 Ehrich[S4]	MCD/NS (pediatric)	8 weeks vs 12 weeks treatment
1999 Bagga[S5]	MCD/NS (pediatric)	8 weeks vs 16 weeks treatment
2000 Hiraoka[S6]	MCD/NS (pediatric)	Lower starting dose
2012 Mishra[S7]	MCD/NS (pediatric)	12 weeks vs 20 weeks treatment
2014 Paul[S8]	MCD/NS (pediatric)	8 weeks vs 12 weeks after achieving remission
2018 Al Talhi[S9]	MCD/NS (pediatric)	Faster taper and total duration 3.5 months vs 6 months
2020 Borovitz[S10]	MCD/NS (pediatric)	Lower starting dose
2021 Kainth[S11]	MCD/NS (pediatric)	2 weeks vs 4 weeks treatment once remission achieved
2021 Sheikh[S12]	MCD/NS (pediatric)	½ starting dose
2022 Mantan[S13]	MCD/NS (pediatric)	Lower dose once remission achieved
2006 Miao[S14]	MCD/NS (adults)	Tacrolimus with ½ prednisone starting dose
2010 Eguchi[S15]	MCD/NS (adults)	Cyclosporin with 0.8 prednisone starting dose
2017 Li[S16]	MCD/NS (adults)	Tacrolimus monotherapy
2018 Remy[S17]	MCD/NS (adults)	Mycophenolic acid with ½ dose starting prednisone
2019 Patil[S18]	MCD/NS (adults)	Tacrolimus monotherapy
2020 Ma[S19]	MCD/NS (adults)	Mycophenolic acid with ¼ dose starting prednisone
2020 Medjeral-Thomas[S20]	MCD/NS (adults)	Tacrolimus monotherapy
2021 Chin[S21]	MCD/NS (adults)	Tacrolimus with ½ prednisone starting dose
2017 Jayne[S22]	AAV	Avacopan no prednisone<br>Avacopan low prednisone<br>High prednisone
2020 Walsh[S23]	AAV	Lower starting dose and faster taper
2021 Jayne[S24]	AAV	Avacopan with rapid prednisone withdrawal
2021 Furuta[S25]	AAV	Lower starting dose and faster taper
1994 Sesso[S26]	LN	IV cyclophosphamide monthly vs IV methylprednisolone
2011 Zeher[S27]	LN	Lower starting dose (0.5mg/kg)
2014 Galbraith[S28]	LN	Removal of maintenance prednisone
2019 Bharati[S29]	LN	Lower starting dose (0.5mg/kg)
2021 Bandhan[S30]	LN	Lower starting dose (0.5mg/kg) and faster taper
2017 Hou[S31]	IgAN	Mycophenolate with ½ prednisone starting dose
2017 Min[S32]	IgAN	Leflunomide with 0.8 prednisone starting dose and shorter duration
2021 Ni[S33]	IgAN	Leflunomide with 2/3 prednisone starting dose

2022 Li[S34]	IgAN	IV methylprednisolone months 0 and 3 with low dose prednisone
2025 Usui[S35]	Membranous nephropathy	Cyclosporin vs Cyclosporin with prednisone 15mg/day

Abbreviations: AAV anti-neutrophil cytoplasm antibody associated vasculitis; GC glucocorticoid; IgAN immunoglobulin A nephropathy; LN lupus nephritis; MCD/NS minimal change disease nephrotic syndrome

Supplementary references

Pediatric MCD/NS

S1. Short versus standard prednisone therapy for initial treatment of idiopathic nephrotic syndrome in children. Arbeitsgemeinschaft für Pädiatrische Nephrologie. Lancet. 1988 Feb 20;1(8582):380-3. PMID: 2893190.

S2. Ueda N, Chihara M, Kawaguchi S, Niinomi Y, Nonoda T, Matsumoto J, Ohnishi M, Yasaki T. Intermittent versus long-term tapering prednisolone for initial therapy in children with idiopathic nephrotic syndrome. J Pediatr. 1988 Jan;112(1):122-6. doi: 10.1016/s0022-3476(88)80136-7. PMID: 3335948.

S3. Tejani A, Suthanthiran M, Pomrantz A. A randomized controlled trial of low-dose prednisone and ciclosporin versus high-dose prednisone in nephrotic syndrome of children. Nephron. 1991;59(1):96-9. doi: 10.1159/000186526. PMID: 1944755.

S4. Ehrich JH, Brodehl J. Long versus standard prednisone therapy for initial treatment of idiopathic nephrotic syndrome in children. Arbeitsgemeinschaft für Pädiatrische Nephrologie. Eur J Pediatr. 1993 Apr;152(4):357-61. doi: 10.1007/BF01956754. PMID: 8482290.

S5. Bagga A, Hari P, Srivastava RN. Prolonged versus standard prednisolone therapy for initial episode of nephrotic syndrome. Pediatr Nephrol. 1999 Nov;13(9):824-7. doi: 10.1007/s004670050708. PMID: 10603129.

S6. Hiraoka M, Tsukahara H, Haruki S, Hayashi S, Takeda N, Miyagawa K, Okuhara K, Suehiro F, Ohshima Y, Mayumi M. Older boys benefit from higher initial prednisolone therapy for nephrotic syndrome. The West Japan Cooperative Study of Kidney Disease in Children. Kidney Int. 2000 Sep;58(3):1247-52. doi: 10.1046/j.1523-1755.2000.00279.x. PMID: 10972687.

S7. Mishra OP, Thakur N, Mishra RN, Prasad R. Prolonged versus standard prednisolone therapy for initial episode of idiopathic nephrotic syndrome. J Nephrol. 2012 May-Jun;25(3):394-400. doi: 10.5301/jn.5000016. PMID: 21928226.

S8. Paul SK, Muinuddin G, Jahan S, Begum A, Rahman MH, Hossain MM. Long versus standard initial prednisolone therapy in children with idiopathic nephrotic syndrome. Mymensingh Med J. 2014 Apr;23(2):261-7. PMID: 24858152.

S9. Al Talhi A, Al Saran K, Osman ET, Al Shatri A, Osman M, Mirza K. A randomized study on a 3-month versus a 7-month prednisolone regimen for the initial episode of childhood idiopathic nephrotic syndrome at a large Saudi center. Int J Pediatr Adolesc Med. 2018 Mar;5(1):18-23. doi: 10.1016/j.ijpam.2017.12.004. Epub 2018 Feb 18. PMID: 30805527; PMCID: PMC6363257.

S10. Borovitz Y, Alfandary H, Haskin O, Levi S, Kaz S, Davidovits M, Dagan A. Lower prednisone dosing for steroid-sensitive nephrotic syndrome relapse: a prospective randomized pilot study. Eur J Pediatr. 2020 Feb;179(2):279-283. doi: 10.1007/s00431-019-03506-5. Epub 2019 Nov 14. PMID: 31728673.

S11. Kainth D, Hari P, Sinha A, Pandey S, Bagga A. Short-Duration Prednisolone in Children with Nephrotic Syndrome Relapse: A Noninferiority Randomized Controlled Trial. Clin J Am Soc Nephrol. 2021 Feb 8;16(2):225-232. doi: 10.2215/CJN.06140420. Epub 2021 Jan 21. PMID: 33478976; PMCID: PMC7863637.

S12. Sheikh S, Mishra K, Kumar M. Low-dose versus conventional-dose prednisolone for nephrotic syndrome relapses: a randomized controlled non-inferiority trial. Pediatr Nephrol. 2021 Oct;36(10):3143-3150. doi: 10.1007/s00467-021-05048-1. Epub 2021 Apr 16. PMID: 33861375.

S13. Mantan M, Kansal A, Swarnim S. Effectiveness of a Low Dose Prednisolone Regimen for Treatment of Relapses in Children with Steroid Sensitive Nephrotic Syndrome. Indian J Nephrol. 2022 Nov-Dec;32(6):588-594. doi: 10.4103/ijn.ijn_463_21. Epub 2022 Jul 16. PMID: 36704589; PMCID: PMC9872916.

Adult MCD/NS

S14. Miao L, Sun J, Yuan H, Jia Y, Xu Z. Combined therapy of low-dose tacrolimus and prednisone in nephrotic syndrome with slight mesangial proliferation. Nephrology (Carlton). 2006 Oct;11(5):449-54. doi: 10.1111/j.1440-1797.2006.00667.x. PMID: 17014560.

S15. Eguchi A, Takei T, Yoshida T, Tsuchiya K, Nitta K. Combined cyclosporine and prednisolone therapy in adult patients with the first relapse of minimal-change nephrotic syndrome. Nephrol Dial Transplant. 2010 Jan;25(1):124-9. doi: 10.1093/ndt/gfp422. Epub 2009 Sep 9. PMID: 19740915.

S16. Li X, Liu Z, Wang L, Wang R, Ding G, Shi W, Fu P, He Y, Cheng G, Wu S, Chen B, Du J, Ye Z, Tao Y, Huo B, Li H, Chen J. Tacrolimus Monotherapy after Intravenous Methylprednisolone in Adults with Minimal Change Nephrotic Syndrome. J Am Soc Nephrol. 2017 Apr;28(4):1286-1295. doi: 10.1681/ASN.2016030342. Epub 2016 Nov 2. PMID: 27807213; PMCID: PMC5373446.

S17. Rémy P, Audard V, Natella PA, Pelle G, Dussol B, Leray-Moragues H, Vigneau C, Bouachi K, Dantal J, Vrigneaud L, Karras A, Pourcine F, Gatault P, Grimbert P, Ait Sahlia N, Moktefi A, Daugas E, Rigothier C, Bastuji-Garin S, Sahali D; MSN Trial Investigators. An open-label randomized controlled trial of low-dose corticosteroid plus enteric-coated mycophenolate sodium versus standard corticosteroid treatment for minimal change nephrotic syndrome in adults (MSN Study). Kidney Int. 2018 Dec;94(6):1217-1226. doi: 10.1016/j.kint.2018.07.021. Epub 2018 Oct 29. PMID: 30385039.

S18. Patil MR, Divyaveer SS, Raychaudhary A, Trivedi M, Mahajan C, Sarkar D, Pandey R. Tacrolimus as the first-line agent in adult-onset minimal change disease: A randomized controlled study. Saudi J Kidney Dis Transpl. 2019 Jan-Feb;30(1):129-137. PMID: 30804274.

S19. Ma MKM, Yap DYH, Li CL, Mok MMY, Chan GCW, Kwan LPY, Lai KN, Tang SCW. Low-dose corticosteroid and mycophenolate for primary treatment of minimal change disease. QJM. 2020 Jun 1;113(6):399-403. doi: 10.1093/qjmed/hcz297. PMID: 31769845.

S20. Medjeral-Thomas NR, Lawrence C, Condon M, Sood B, Warwicker P, Brown H, Pattison J, Bhandari S, Barratt J, Turner N, Cook HT, Levy JB, Lightstone L, Pusey C, Galliford J, Cairns TD, Griffith M. Randomized, Controlled Trial of Tacrolimus and Prednisolone Monotherapy for Adults with De Novo Minimal Change Disease: A Multicenter, Randomized, Controlled Trial. Clin J Am Soc Nephrol. 2020 Feb 7;15(2):209-218. doi: 10.2215/CJN.06180519. Epub 2020 Jan 17. Erratum in: Clin J Am Soc Nephrol. 2020 Jul 1;15(7):1027. doi: 10.2215/CJN.06290420. PMID: 31953303; PMCID: PMC7015084.

S21. Chin HJ, Chae DW, Kim YC, An WS, Ihm C, Jin DC, Kim SG, Kim YL, Kim YS, Kim YG, Koo HS, Lee JE, Lee KW, Oh J, Park JH, Jiang H, Lee H, Lee SK. Comparison of the Efficacy and Safety of Tacrolimus and Low-Dose Corticosteroid with High-Dose Corticosteroid for Minimal Change Nephrotic Syndrome in Adults. J Am Soc Nephrol. 2021 Jan;32(1):199-210. doi: 10.1681/ASN.2019050546. Epub 2020 Nov 9. PMID: 33168602; PMCID: PMC7894664.

ANCA-associated vasculitis

S22. Jayne DRW, Bruchfeld AN, Harper L, Schaier M, Venning MC, Hamilton P, Burst V, Grundmann F, Jadoul M, Szombati I, Tesař V, Segelmark M, Potarca A, Schall TJ, Bekker P; CLEAR Study Group. Randomized Trial of C5a Receptor Inhibitor Avacopan in ANCA-Associated Vasculitis. J Am Soc Nephrol. 2017 Sep;28(9):2756-2767. doi: 10.1681/ASN.2016111179. Epub 2017 Apr 11. PMID: 28400446; PMCID: PMC5576933.

S23. Walsh M, Merkel PA, Peh CA, Szpirt WM, Puéchal X, Fujimoto S, Hawley CM, Khalidi N, Floßmann O, Wald R, Girard LP, Levin A, Gregorini G, Harper L, Clark WF, Pagnoux C, Specks U, Smyth L, Tesar V, Ito-Ihara T, de Zoysa JR, Szczeklik W, Flores-Suárez LF, Carette S, Guillevin L, Pusey CD, Casian AL, Brezina B, Mazzetti A, McAlear CA, Broadhurst E, Reidlinger D, Mehta S, Ives N, Jayne DRW; PEXIVAS Investigators. Plasma Exchange and Glucocorticoids in Severe

ANCA-Associated Vasculitis. N Engl J Med. 2020 Feb 13;382(7):622-631. doi: 10.1056/NEJMoa1803537. PMID: 32053298; PMCID: PMC7325726.

S24. Jayne DRW, Merkel PA, Schall TJ, Bekker P; ADVOCATE Study Group. Avacopan for the Treatment of ANCA-Associated Vasculitis. N Engl J Med. 2021 Feb 18;384(7):599-609. doi: 10.1056/NEJMoa2023386. Erratum in: N Engl J Med. 2024 Jan 25;390(4):388. doi: 10.1056/NEJMx230010. PMID: 33596356.

S25. Furuta S, Nakagomi D, Kobayashi Y, Hiraguri M, Sugiyama T, Amano K, Umibe T, Kono H, Kurasawa K, Kita Y, Matsumura R, Kaneko Y, Ninagawa K, Hiromura K, Kagami SI, Inaba Y, Hanaoka H, Ikeda K, Nakajima H; LoVAS Collaborators. Effect of Reduced-Dose vs High-Dose Glucocorticoids Added to Rituximab on Remission Induction in ANCA-Associated Vasculitis: A Randomized Clinical Trial. JAMA. 2021 Jun 1;325(21):2178-2187. doi: 10.1001/jama.2021.6615. PMID: 34061144; PMCID: PMC8170547.

Lupus Nephritis

S26. Sesso R, Monteiro M, Sato E, Kirsztajn G, Silva L, Ajzen H. A controlled trial of pulse cyclophosphamide versus pulse methylprednisolone in severe lupus nephritis. Lupus. 1994 Apr;3(2):107-12. doi: 10.1177/096120339400300209. PMID: 7920609.

S27. Zeher M, Doria A, Lan J, Aroca G, Jayne D, Boletis I, Hiepe F, Prestele H, Bernhardt P, Amoura Z. Efficacy and safety of enteric-coated mycophenolate sodium in combination with two glucocorticoid regimens for the treatment of active lupus nephritis. Lupus. 2011 Dec;20(14):1484-93. doi: 10.1177/0961203311418269. Epub 2011 Oct 5. PMID: 21976398.

S28. Galbraith L, Manns B, Hemmelgarn B, Walsh M. The Steroids In the Maintenance of remission of Proliferative Lupus nephritis (SIMPL) pilot trial. Can J Kidney Health Dis. 2014 Nov 28;1:30. doi: 10.1186/s40697-014-0030-9. PMID: 25780619; PMCID: PMC4349625.

S29. Bharati J, Rathi M, Ramachandran R, Sharma A, Kumar V, Kohli HS, Gupta KL. Comparison of Two Steroid Regimens in Induction Therapy of Proliferative Lupus Nephritis: A Randomized Controlled Trial. Indian J Nephrol. 2019 Sep-Oct;29(5):373-375. doi: 10.4103/ijn.IJN_299_18. PMID: 31571750; PMCID: PMC6755920.

S30. Bandhan IH, Islam MN, Ahmad HI, Ahmedullah AK. Outcome of low-dose prednisolone use for the induction of remission in lupus nephritis patients. Int J Rheum Dis. 2022 Feb;25(2):121-130. doi: 10.1111/1756-185X.14265. Epub 2021 Dec 11. PMID: 34894070.

IgA nephropathy

S31. Hou JH, Le WB, Chen N, Wang WM, Liu ZS, Liu D, Chen JH, Tian J, Fu P, Hu ZX, Zeng CH, Liang SS, Zhou ML, Zhang HT, Liu ZH. Mycophenolate Mofetil Combined With Prednisone Versus Full-Dose Prednisone in IgA Nephropathy With Active Proliferative Lesions: A Randomized

Controlled Trial. Am J Kidney Dis. 2017 Jun;69(6):788-795. doi: 10.1053/j.ajkd.2016.11.027. Epub 2017 Feb 16. PMID: 28215945.

S32. Min L, Wang Q, Cao L, Zhou W, Yuan J, Zhang M, Che X, Mou S, Fang W, Gu L, Zhu M, Wang L, Yu Z, Qian J, Ni Z. Comparison of combined leflunomide and low-dose corticosteroid therapy with full-dose corticosteroid monotherapy for progressive IgA nephropathy. Oncotarget. 2017 Jul 18;8(29):48375-48384. doi: 10.18632/oncotarget.16468. PMID: 28415636; PMCID: PMC5564655.

S33. Ni Z, Zhang Z, Yu Z, Lu F, Mei C, Ding X, Yuan W, Zhang W, Jiang G, Sun M, He L, Deng Y, Pang H, Qian J. Leflunomide plus low-dose prednisone in patients with progressive IgA nephropathy: a multicenter, prospective, randomized, open-labeled, and controlled trial. Ren Fail. 2021 Dec;43(1):1214-1221. doi: 10.1080/0886022X.2021.1963775. PMID: 34396911; PMCID: PMC8381933.

S34. Li Y, Fu R, Gao J, Wang L, Duan Z, Tian L, Ge H, Ma X, Zhang Y, Li K, Xu P, Tian X, Chen Z. Effect of pulsed intravenous methylprednisolone with alternative low-dose prednisone on high-risk IgA nephropathy: a 18-month prospective clinical trial. Sci Rep. 2022 Jan 7;12(1):255. doi: 10.1038/s41598-021-03691-0. PMID: 34996948; PMCID: PMC8742122.

Membranous nephropathy

S35. Usui J, Hirayama K, Kobayashi M, Suzuki S, Ebihara I, Nishiki K, Mase K, Hirayama A, Saito C, Goto M, Koyama A, Yamagata K. Randomized Prospective Controlled Open-labeled Trial of Cyclosporine with/without Low-dose Oral Corticosteroids in Idiopathic Membranous Nephropathy in Adults with Nephrotic Syndrome. Intern Med. 2025 Sep 1;64(17):2541-2548. doi: 10.2169/internalmedicine.4803-24. Epub 2025 Feb 22. PMID: 39993753; PMCID: PMC12463419.
